# Taoist way of a balanced exercise training cocktail for the management of primary hypertension in older persons

**DOI:** 10.3389/fpubh.2023.1308375

**Published:** 2023-12-07

**Authors:** Wensheng Xiao, Bihan Wang, Xiaorong Bai, Shouyong Tang, Yang Zhang

**Affiliations:** ^1^School of Physical Education, Huzhou University, Huzhou, China; ^2^College of Physical Education, Hunan Normal University, Changsha, China; ^3^Institute of Sports and Health Industry, HEHA CAT Fitness, Changsha, China; ^4^Independent Person, Windermere, FL, United States

**Keywords:** balance, blood pressure, falls, physical activity, meta-analysis, Tai Chi, Tao

## Abstract

High blood pressure is the world’s leading risk factor for mortality, affecting nearly half of the global population aged 50–79 years. Physical inactivity is one factor contributing to the prevalence of hypertension. This paper discusses a new concept for the management of hypertension in older persons. We are inclined to fade the current guidelines used in China, the United States, and Europe. Although demonstrating irrefutable benefits for blood pressure regulation, the guidelines fail to address the need to incorporate balance exercises, which are crucial for mitigating the risk of falling. We address three pressing questions regarding the efficacy of various combinations of exercise modes for blood pressure regulation, alongside providing an overview of balance exercises. At the core of our concept, we explicate the challenges inherent in addressing the global pandemic of physical inactivity and hypertension in regular socioeconomic people. No guidelines could change the state of inactivity by jumping between zero and all things, where “zero” symbolizes conditions such as physical inactivity and hypertension, and the concept of “all things” encompasses the ideals of an active lifestyle and healthy aging. We advocate a Taoist way, “zero–one—all things,” where “one” in this context refers to an inclusive and culturally diverse exercise training cocktail. The Tao guides us to illuminate an ancient way of overcoming physical inactivity-associated diseases in the present day.

## 1 Introduction

Dating back to the Warring States Period, the Esoteric Scripture of the Yellow Emperor made observations regarding the impact of an irregular pulse on human health (1). The scripture noted that over-consumption of salt might lead to impaired blood circulation and subsequently impact one’s facial complexion (“多食鹹則脈凝泣而變色”), and that those who consume excessive amounts of salt and experience heart failure may exhibit an increased and hardened pulse (“[脈]盛而緊曰脹”). These vivid writings may constitute the earliest documentation of primary hypertension (hereafter, hypertension), suggesting that hypertension is not a recent ailment. Nevertheless, as we learn from the oracle bone script and beyond, securing a sufficient calorie intake was the paramount daily concern for the ancient Chinese, and the population halved every few hundred years as a result of famine and revolt (2). Obesity, insulin resistance, high alcohol intake, aging, or sedentary lifestyles are all recent “luxury” causes of hypertension (3) that have emerged in industrial societies, making hypertension a modern disease.

In 2019, approximately 1.3 billion of the global population aged 30–79 years were living with hypertension (4). Hypertension has emerged as the primary risk factor for early mortality on a global scale, resulting in an estimated annual count of 10.8 million avoidable deaths (5). During the 78th Session of the United Nations General Assembly, the World Health Organization officially launched global efforts to prevent and cure this global disease, which it has referred to as “the race against a silent killer” (6). The worldwide hypertensive population will continually trend higher in the absence of lifestyle modifications, among which the level of physical activity plays a crucial role in the prevention–development–management nexus of high blood pressure (BP).

To date, there is conclusive evidence that physical inactivity is an etiology of hypertension and, conversely, regular physical activity (hereafter, exercise training) is an important adjuvant to standard pharmacological treatments (7). Despite its exceptional preventive and therapeutic values, the global level of physical activity has been on a downward trend and accelerated throughout the height of the COVID-19 pandemic (8). Should the existing prevalence of physical inactivity remain unchanged, a staggering 240 million new cases of preventable hypertension are projected to emerge worldwide between 2020 and 2030, which will impose a more than 115 billion US dollar burden on public healthcare systems (9). This polarization between evidence and action can be ascribed to myriad problems (10), but the elephant in the room is the lack of an all-round exercise training recommendation considered for regular socioeconomic people.

In this perspective, we first provide an overview of the existing exercise training guidelines for treating hypertension, with a focus on older persons. Old age is an independent risk factor of hypertension. On a global scale, the prevalence of hypertension among adults aged 30–79 years is estimated to be 33%, with a notable rise to 49% among those aged 50 to 79 (11). With the global demographic shift towards an aging society, hypertension will impose an even greater burden on healthcare systems. The aim of this discussion is to illuminate a way towards all-round exercise training guidelines for regular socioeconomic people with hypertension.

## 2 Exercise guidelines

The lack of policy emphasis on physical activity has been subject to criticism for impeding the successful implementation of evidence-based exercise training programs (10). But it is also important to be honest about the scale of the challenge. Part of the reason for hypertensive patients’ current condition is a lack of exercise training. This further exacerbates the difficulties associated with engaging in exercise training when they are well past the stage of developing habitual active living habits, from both a physiological and psychological perspective. Meanwhile, individuals diagnosed with hypertension tend to have an increased susceptibility to comorbidities, such as the progressive development of cardiovascular diseases, diabetes mellitus, and renal diseases (12, 13). Sub-optimal health circumstances considerably limit options for participating in highly structured exercise training programs. Furthermore, the effectiveness of exercise training is significantly influenced by exercise adherence, with ample evidence confirming that acceptable adherence rates are achieved only in short-term, supervised clinical settings (14).

**Figure 1:**
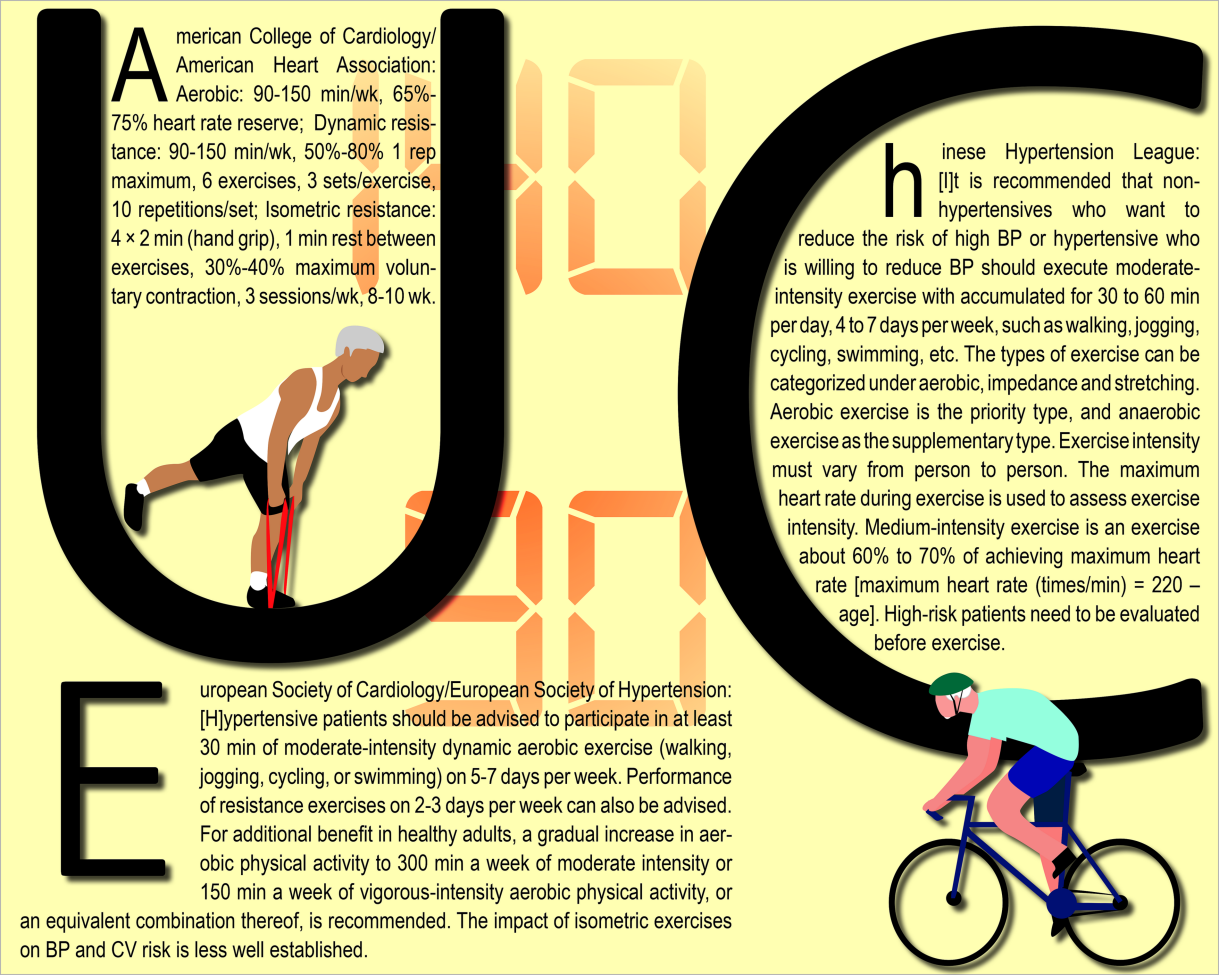
Featured exercise training guidelines for the management of hypertension.

With the aforementioned practical barriers in mind, we will examine three exercise training guidelines that are currently recommended for hypertensive patients in China, the United States, and Europe (15–17). The guidelines, illustrated in Figure 1, all advocate the inclusion of both aerobic and resistance exercises on a regular basis, typically spanning most days of the week, as an effective approach to reducing BP. American institutions value both aerobic and resistance exercise equally, while Chinese and European institutions prioritize aerobic exercise, with additional benefits derived from incorporating resistance exercise. In addition, the American guidelines suggest adding isometric resistance, which is a well-founded recommendation given that there is a significant correlation between a reduction in handgrip strength and the occurrence of old-age disabilities (18). These guidelines are backed by strong clinical evidence; therefore, it is not unexpected that they share similarities. In the meantime, we are inclined to suggest that they also reflect the trend of following the academic zeitgeist.

In the current context, domain-theory-driven guidelines demonstrate conceptual rigor but are somewhat impractical in real-life situations—it is as if we expect conscientious hypertensive patients to constantly evaluate their health status and activity requirements, and then factor in this information to their daily decision-making process. In everyday life, regular socioeconomic people often do not grasp the concept of periodic exercise training (19) and they may struggle to understand scientific methodology explained in specialized terminology. Indeed, there are even academic debates surrounding the technical language used in the assessment and prescription of exercise intensity, prompting the European Association of Preventive Cardiology to provide clarification and standardization of the terminology used (20). Moreover, as numerous experiences have indicated, once individuals transition from in-person supervised exercise training to the home environment, long-term exercise adherence cannot be maintained. All of these factors leave a practical gap between knowledge of the benefits of exercise and the behavioral changes required for maintaining exercise training among the hypertensive population.

The above critique is not a new point of view. The current physical activity guidelines of the World Health Organization recommend that all persons aged over 65 years engage in multi-component physical activity that prioritizes functional balance and strength training (21). Our recent meta-analysis further reviews evidence of the cellular advantages associated with multi-component exercise training and its implications for extending the healthspan (22). Therefore, the true deficiency in the existing guidelines for the management of hypertension is the lack of specific multi-component exercise training, as well as the absence of any reference to an exercise countermeasure to address a significant risk factor for premature mortality—accidental falls among older persons. The resultant gap is a small deficiency in exercise training modes, and a seemingly “little” accident can lead to a much higher probability of older persons losing their ability to live independently or may even lead to them dying.

Falls represent the leading cause of injuries among older persons in China (23) and the United States (24), with the resulting injuries strongly contributing to complications and death within this population globally (25). Aging and hypertension often coexist, leading to a higher incidence of falls. Inconveniently, the use of anti-hypertensive medication in older persons may amplify the risk of falling. A cautious interpretation of longitudinal studies points to hypertension exacerbating age-associated fall risks, the initiation of anti-hypertensive drugs (26, 27) may further expose individuals to such risks, and the intensification of anti-hypertensive drug usage (26, 28) may heighten the long-term risk of falling in older patients. While exercise-induced reduction in BP is reported to reduce all-cause mortality by 4% (29), which is a welcome development, the obvious concern is that a single severe fall could end many efforts at staying healthy, if not an older person’s life. From a practical perspective, none of the aforementioned exercise training guidelines for hypertension provide a complete recipe for success in the pursuit of healthy aging. To our knowledge, only the recent revision of the International Society of Hypertension’s guideline mentions the need for additional flexibility and balance exercise (30). Nevertheless, neither the infographic intended for public consumption nor the technical description provide an evidence-based approach toward balance exercise. Consequently, there is an enormous gap in existing anti-hypertensive exercise training guidelines, necessitating the inclusion of multi-component activities with special consideration to balance exercise for older persons.

## 3 Remixing meta-analyses

According to the World Health Organization (21), multi-component exercise training involves “combinations of balance, strength, endurance, gait, and physical function training.” When expressed in strict, straightforward terms, whether the existing guidelines (15–17, 30) can achieve comparable BP benefits to concurrent aerobic and resistance exercise is in some respects debatable, but the overarching point is that these guidelines do not explicitly mention the inclusion of multi-component exercise modes within the same session, and nor do they address the aspect of balance exercise. Hence, multi-component exercise training in this perspective can be operationally defined as a structured exercise session that integrates aerobic, resistance, and balance exercises with the aim of reducing BP and enhancing balance. Ideally, multi-component activities could concurrently improve flexibility. To provide a concise overview of scientific evidence on exercise training outcomes, we examined updated meta-analyses and will present a few representative studies published by leading academic societies. Three research questions are retrospectively examined.

Question 1: Which type of exercise training is better in terms of BP control? When combined with hypertension medication, aerobic exercise could significantly reduce daytime systolic and diastolic BP by −5 and −3.5 mmHg, respectively (31); resistance exercise could significantly reduce daytime systolic and diastolic BP by −4.7 and −3.5 mmHg, respectively (32); and isometric resistance exercise could significantly reduce daytime systolic and diastolic BP by −7.5 and −3.2 mmHg, respectively (33). Since previous meta-analyses of multi-component exercise training for the treatment of hypertension only covered a small amount of the available literature (*n* = 2) (31) or examined its effect on systolic BP only (34), we conducted an updated one (*n* = 7) and present the results here (see also the Supplementary Material for detailed methods: https://doi.org/10.6084/m9.figshare.24247861.v1). Based on empirical evidence from older persons with hypertension, multi-component exercise training could significantly reduce daytime systolic and diastolic BP by −9.5 and −6.7 mmHg, respectively (35–41). Of note, high-intensity interval training is not taken into consideration in our perspective due to the lack of evidence supporting its long-term adherence among older persons. Taken collectively, multi-component exercise training appears to offer additional benefits in controlling BP compared to aerobic or resistance exercise training alone.

Question 2: Does exercise training improve balance or reduce the risk of falling in older persons with hypertension? This question is difficult to answer for two reasons. First, the current guidelines (15–17) overlook the need of including balance exercise in exercise training programs for older hypertensive patients, and it is evident that balance assessment was seldom included as an outcome measure in the relevant literature. Second, there is no universally accepted diagnostic criterion for assessing the risk of falling. Several assessments, such as the one-leg standing test (42) and the timed up and go test (43), have acceptable discriminatory ability in identifying the risk of falling among low-functioning older persons. With these shortcomings in mind, we undertook a subsequent meta-analysis, specifically addressing multi-component exercise training (see also Supplementary Material). Based on the available evidence (*n* = 4) (35, 39, 40, 44), the results of the timed up and go test indicate a difference of −0.26 s (95% CI −0.66 to 0.14) between the multi-component exercise training group and the no exercise training group. In simpler terms, a combination of aerobic and resistance exercise (39, 40), or aerobic and functional exercise (35, 44), did not improve balance or reduce fall risks. Our cautious takeaway is that multi-component exercise training, in the absence of a dedicated balance exercise, is unable to effectively achieve both BP control and balance improvement simultaneously.

Question 3: What do empirical data suggest about the efficacy of exercise modes in enhancing balance among older persons in general? To ease the promotion of balance exercises, we introduce approaches with the potential to serve as a feasible framework transcending ethnoses and socioeconomic boundaries, and we interpret the academic literature using the common-language effect size (45). The simplest form of linear movement approach—Nordic walking—has been shown to have a 58.4% chance of improving dynamic balance (timed up and go test) in healthy older persons (46). Backward walking has been found to enhance static balance (one-leg standing test) by 0.91 s in healthy older persons (47), equivalent to an approximate 5% decrease in the likelihood of experiencing hip fractures (48). Although such odds seem like a coin toss, it is important to note that walking has the potential to be the most widely practiced activity for preventing falls in an unsupervised home environment (49). Stepping exercises can be implemented to enhance the complexity of gait training in a two-dimensional manner, and this approach has been found to have a 61.4% chance of enhancing dynamic balance (timed up and go test) in older persons (50). When it comes to 3D-type balance exercises, Pilates and yoga are popular exercises among women. Pilates training has been found to have a 70.2% chance of enhancing dynamic balance (timed up and go test) (51), while yoga has been found to have a 67.5% chance of enhancing overall balance (52) in healthy older persons. In the last two decades, the health and therapeutic benefits of Taijiquan (commonly known as Tai Chi) has garnered more acknowledgment beyond China (53, 54). Research conducted on older persons found that engaging in Taijiquan practice improved static balance (one-leg standing test) by an average of 5.33 s (55), and has been found to be associated with a 20 and 31% reduction in the risk of falling and rate of falls, respectively (56). It is worth mentioning that Taijiquan practice as a part of anti-hypertensive intervention could significantly reduce daytime systolic and diastolic BP by −12.5 and − 6.5 mmHg, respectively (57), which highlights its origins as a martial art. It is said that “They do not hurt each other, and the virtue in each one refreshes both” (Tao Te Ching, Chapter 60). We believe that practicing any of the aforementioned balance exercises will be beneficial for older persons in the long term and two is greater than one.

## 4 Zero, one, then all things

In 485 BCE, prior to Lao Tzu embarking on his final journey into the wilderness, he composed Tao Te Ching, wherein he articulated the Tao ideas: “The Tao begot one. One begot two. Two begot three. And three begot the ten thousand things.” More than a decade ago, when I asked my mentor (Phil Bishop, Emeritus Professor of the University of Alabama) for his advice on recommended exercise for weight loss, his response was succinct: “move first.” By combining contemporary insight with ancient wisdom, we advocate for a holistic approach to promote physical activity — “zero, one, then all things”. Esoteric though it may be, this is the natural sequence, especially now, as academics ponder the growing contrast between their sound scientific exercise prescriptions and the pandemic of physical inactivity (10). Too often, overly prescribed exercise intensity has actually been a source of muted positive affective responses (58), particularly among sedentary individuals (59)—indeed, it has been a sponsor of low exercise adherence.

With apologies to the billions of wonderful people on Earth, let the authors portray the realities facing regular socioeconomic people. These people are usually not inspired by academic jargon. They are often exposed to cheap, low-nutrient dense calories (60) and are often engaged in physically light duties. They have the highest marginal propensity to essential consumption. Moreover, they are mostly insulated from fiscal largesse. These factors are all ominous for the race against physical inactivity.

For the Taoists, such a non-movement, abstract being state can be referred to as zero, the formless essence of the Universe at the beginning. In contrast, exercise training can be viewed as a formed state. The current guidelines on one step fixing the “zero” overlook the natural developmental process where “infants” learn to walk before they can run.

The Tao begot one, the change leading to order and then forming all things. One in Tao is seen as a symbol of water, with the ability to exhibit both gentle and gradual movements, as well as the capacity to erode even the most resilient of rocks over time. Above all, water is esteemed for its greatest virtue of fostering life and sustaining the existence of all things. The sooner people accept that the one is the missing force for differentiation between undifferentiated non-movement and differentiated being, the sooner the world can begin to bridge the deep divisions between zero and all things.

**Figure 2:**
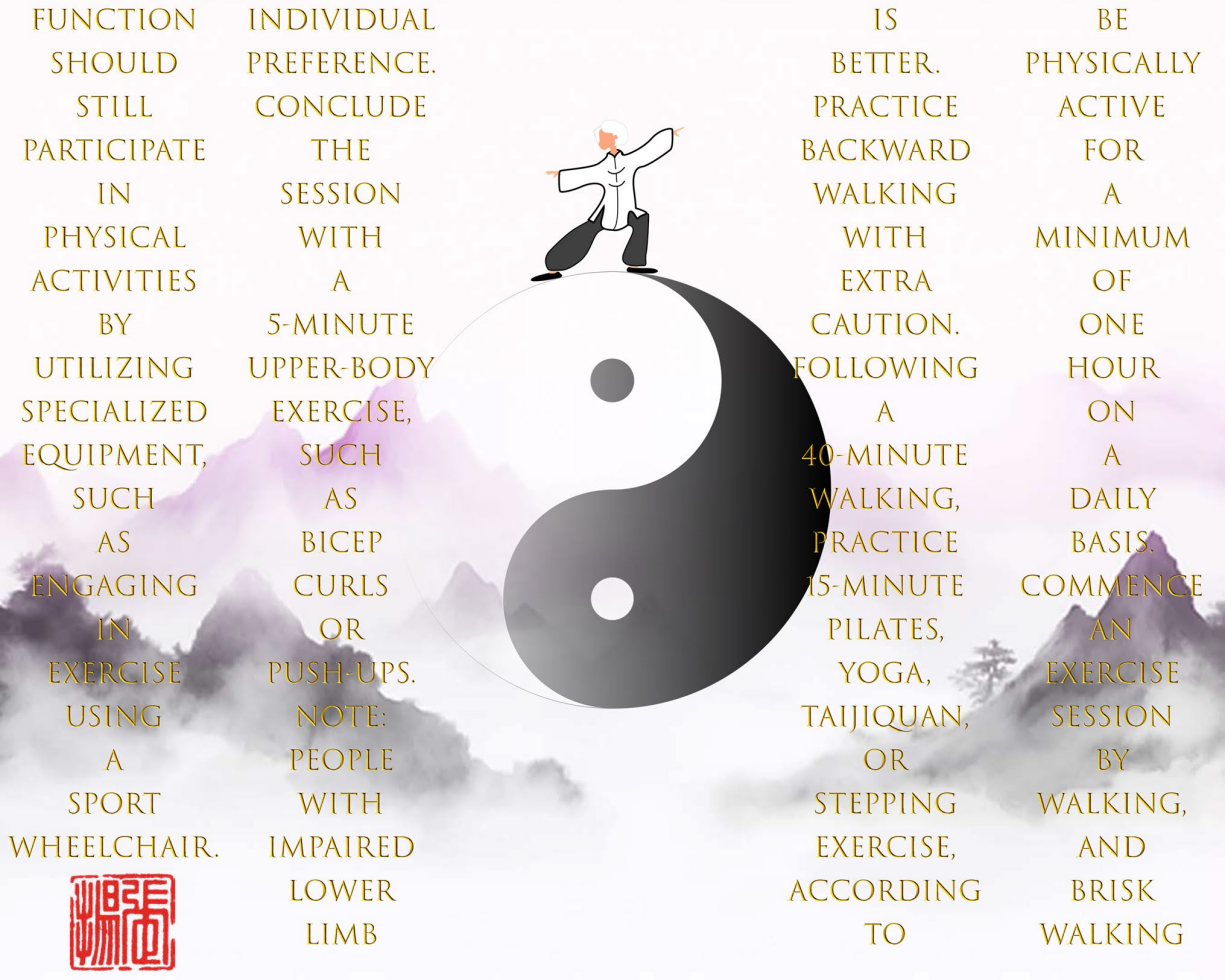
Taoist’s exercise training concept — zero, one, then all things. Note: Texts should be read from top to bottom and from right to left.

Although the emphasis in the current guidelines on effective BP-reducing workouts is sound, albeit incomplete in our view, society is not ready for the swift adoption of the structured exercises prescribed in academic jargon. There must be a clearly defined equilibrium status between the present state and the ideal level of physical activity. In Figure 2, we describe the essential “one,” a cocktail of walking, balance exercises, and upper-body exercise as a life-awakening program for hypertensive patients. To begin with, walking serves as the fundamental basis for all-round exercise training. Walking is the most convenient form of aerobic exercise for older persons who do not have severe physical limitations. Walking not only decreases BP (61, 62) but also enhances lower limb strength (63), hence reducing the risk of falling (64) and all-cause mortality (65). Thus, it functions as a holistic means of managing hypertension, balance, and lifespan for older persons.

Modern medicine has demonstrated the excellent efficacy and low toxicity of drugs used in the management of high BP (66). From a therapeutic standpoint, exercise intervention is arguably an adjuvant endeavor. Put differently, hypertensive patients can live with this medical condition without engaging in exercise training, notwithstanding the myriad of physiological and financial benefits obtained from exercise. Nevertheless, the fact that there is no pill targeting age-associated decline in balance control underscores the significance of the balance exercise component in our way. As mentioned above, there are various culturally diverse exercises for developing balance control. Our recent randomized controlled experiment demonstrated that a multi-component exercise training program, comprising walking and Taijiquan, can enhance static balance, as well as improve flexibility, handgrip strength, and lower limb endurance in normotensive older persons (67). Hypertensive patients within the Chinese cultural radius thus can choose to practice a simplified form of Taijiquan, which typically lasts for approximately 15 min, to improve their balance control. Although new work is necessary to validate the effectiveness of a multi-component exercise training program that includes walking and yoga, Pilates, or stepping exercises for controlling BP and balance control, our assertion of the importance of balance exercise within a comprehensive BP management strategy is hard to ignore. Thus, incorporating diverse balance exercises is the essence of a balanced exercise training program for hypertensive patients as they age.

Incorporating a resistance exercise component not only contributes to the hierarchical nature of BP-reducing exercise training, but also adds to the prospects of improved lifespan outcomes. A previous cohort study compared various forms of exercise and sports, concluding that engagement in racquet sports exhibited the most significant reduction in all-cause and cardiovascular diseases mortality (68). A potential explanation may be that racquet sports incorporate whole-body movements, aligning with the conclusions of our recent meta-analysis (22). Briefly, training a wide range of muscle groups can enhance the expression of intracellular nicotinamide phosphoribosyltransferase, leading to increased levels of nicotinamide adenine dinucleotide during the aging process. This in turn could aid older persons in protecting themselves against environmental insults and enhance BP management in people with hypertension (69). Nevertheless, we do not explicitly recommend hypertensive patients to engage in racquet sports as a form of upper-body exercise. From our perspective, regular socioeconomic people lack the necessary resources, such as finances and time, to participate in racquet sports on a daily basis. As a feasible alternative, we suggest incorporating straightforward yet effective upper-body exercises to encourage and establish exercise adherence.

None of the existing guidelines (15–17, 30) include targeted exercise recommendations for individuals with impaired lower limb function or disability, which is another critical deficiency. Our way first seeks to extend the boundaries of exercise training guidelines and includes special sociodemographic populations. Although exercise equipment such as an arm crank ergometer could address the mobility dilemma, we explicitly recommend using a sports wheelchair as an optimal choice for exercise training. Despite both the arm crank ergometer and sports wheelchair having the potential to provide similar physiological benefits when handled correctly, the key distinction lies in the exercise environment. Engaging in exercise training in calm outdoor environments, such as walking, using a sports wheelchair, or practicing Taijiquan in a park, can enhance sensations of vigor, which in turn have a positive impact on mental wellbeing and exercise attendance and adherence (70). Finally, although poor mobility or disability should not be used as a justification for physical inactivity, it is important to acknowledge the lack of awareness, knowledge, and financial challenges experienced by regular socioeconomic people. To address this issue, it is necessary to establish a collaborative governance system where the government takes the lead in providing fiscal support and mobilizing society forces (71). This would enable the dissemination of instructional materials and the co-funding of affordable products to fulfill the specific market demands.

One notable feature of the Taoist way is its potential to achieve a harmonious blend of scientific principles and sustainability. Using walking as a focal point, our way does not specify the pace of walking, as empirical data align with the hedonic theory and support the use of self-paced intensity to promote exercise adherence, especially if individuals are not used to being physically active (72). While it may seem that engaging in a 45-min self-paced walking activity may not meet the recommended thresholds for reducing the risk of falling (5,000 steps per day) (64) and all-cause mortality (6,000–8,000 steps per day) (65), it is essential to not to lose the sight of the fact that our way aims to cultivate a disciplined active lifestyle among sedentary individuals in the later stages of life. After following the “one” for at least a year, hypertensive patients are advised to progressively incorporate additional scientific guidelines (15–17) as needed, which represents the second stage of the formed state. Ultimately, Tao resists objectification yet is immanent in the way cultivated people spontaneously engage in physical activity and generate health benefits.

## 5 Conclusion

Tao is the way and science is the light, from which we project ourselves into the global situation and form this guide. It will serve as an impetus for further discussion of the early prevention of noncommunicable diseases and robust systems of governance for population fitness (71). It is said that “Under heaven nothing is more soft and yielding than water. Yet for attacking the solid and strong, nothing is better.” Overall, we salute the ancient wisdom by orienting a multi-component exercise training cocktail, featuring inclusive sociodemographic populations and culturally diverse balance exercises for the management of hypertension.

“The ten thousand things carry yin and embrace yang. They achieve harmony by combining these forces.” — Lao Tzu, Chapter 42 of Tao Te Ching

## Data availability statement

The original contributions presented in the study are included in the article/supplementary materials, further inquiries can be directed to the corresponding author.

## Author contributions

WX: Data curation, Formal analysis, Writing – original draft. BW: Validation, Writing – original draft. XB: Data curation, Formal analysis, Writing – original draft. ST: Writing – original draft. YZ: Conceptualization, Methodology, Project administration, Supervision, Visualization, Writing – original draft, Writing – review & editing.

## References

[1] Kalehoff JP Oparil S. The story of the silent killer. Curr Hypertens Rep. (2020) 22:72. doi: 10.1007/s11906-020-01077-7, PMID: 32852612

[2] Chu CYC Lee RD. Famine, revolt, and the dynastic cycle. J Popul Econ. (1994) 7:351–78. doi: 10.1007/BF00161472, PMID: 12288504

[3] Carretero OA Oparil S. Essential Hypertension. Circulation. (2000) 101:329–35. doi: 10.1161/01.CIR.101.3.329 10645931

[4] Zhou B Carrillo-Larco RM Danaei G Riley LM Paciorek CJ Stevens GA. Worldwide trends in hypertension prevalence and progress in treatment and control from 1990 to 2019: a pooled analysis of 1201 population-representative studies with 104 million participants. Lancet. (2021) 398:957–80. doi: 10.1016/S0140-6736(21)01330-1, PMID: 34450083 PMC8446938

[5] Murray CJL Aravkin AY Zheng P Abbafati C Abbas KM Abbasi-Kangevari M. Global burden of 87 risk factors in 204 countries and territories, 1990–2019: a systematic analysis for the global burden of disease study 2019. Lancet. (2020) 396:1223–49. doi: 10.1016/S0140-6736(20)30752-2, PMID: 33069327 PMC7566194

[6] World Health Organization (2023). Global report on hypertension: The race against a silent killer. World Health Organization. Available at: https://www.who.int/teams/noncommunicable-diseases/hypertension-report [Accessed September 19, 2023].

[7] Olsen MH Angell SY Asma S Boutouyrie P Burger D Chirinos JA. A call to action and a lifecourse strategy to address the global burden of raised blood pressure on current and future generations: the lancet commission on hypertension. Lancet. (2016) 388:2665–712. doi: 10.1016/S0140-6736(16)31134-5 27671667

[8] Amini H Habibi S Islamoglu AH Isanejad E Uz C Daniyari H. COVID-19 pandemic-induced physical inactivity: the necessity of updating the global action plan on physical activity 2018-2030. Environ Health Prev Med. (2021) 26:32. doi: 10.1186/s12199-021-00955-z, PMID: 33678154 PMC7937363

[9] Santos AC Willumsen J Meheus F Ilbawi A Bull FC. The cost of inaction on physical inactivity to public health-care systems: a population-attributable fraction analysis. Lancet Glob Health. (2023) 11:e32–9. doi: 10.1016/S2214-109X(22)00464-8, PMID: 36480931 PMC9748301

[10] Pratt M Varela AR Salvo D III Ding D. Attacking the pandemic of physical inactivity: what is holding us back? Br J Sports Med. (2020) 54:760–2. doi: 10.1136/bjsports-2019-101392, PMID: 31704698

[11] Zhou B Perel P Mensah GA Ezzati M. Global epidemiology, health burden and effective interventions for elevated blood pressure and hypertension. Nat Rev Cardiol. (2021) 18:785–802. doi: 10.1038/s41569-021-00559-8, PMID: 34050340 PMC8162166

[12] Krolewski AS Canessa M Warram JH Laffel LMB Christlieb R Knowler WC. Predisposition to hypertension and susceptibility to renal disease in insulin-dependent diabetes mellitus. N Engl J Med. (1988) 318:140–5. doi: 10.1056/nejm198801213180303 3336401

[13] Tatsumi Y Ohkubo T. Hypertension with diabetes mellitus: significance from an epidemiological perspective for Japanese. Hypertens Res. (2017) 40:795–806. doi: 10.1038/hr.2017.67 28701739

[14] Lopes S Félix G Mesquita-Bastos J Figueiredo D Oliveira J Ribeiro F. Determinants of exercise adherence and maintenance among patients with hypertension: a narrative review. Rev Cardiovasc Med. (2021) 22:1271–8. doi: 10.31083/j.rcm2204134, PMID: 34957769

[15] Whelton PK Carey RM Aronow WS Casey DE Collins KJ Himmelfarb CD. 2017 ACC/AHA/AAPA/ABC/ACPM/AGS/APhA/ASH/ASPC/NMA/PCNA guideline for the prevention, detection, evaluation, and Management of High Blood Pressure in Adults. J Am Coll Cardiol. (2018) 71:e127–248. doi: 10.1016/j.jacc.2017.11.006, PMID: 29146535

[16] Williams B Mancia G Spiering W Agabiti Rosei E Azizi M Burnier M. 2018 ESC/ESH guidelines for the management of arterial hypertension: the task Force for the management of arterial hypertension of the European Society of Cardiology (ESC) and the European Society of Hypertension (ESH). Eur Heart J. (2018) 39:3021–104. doi: 10.1093/eurheartj/ehy339, 30165516

[17] LIU LS JCfG R. Chinese guidelines for prevention and treatment of hypertension—A report of the revision Committee of Chinese Guidelines for prevention and treatment of hypertension. J Geriatr Cardiol. (2018, 2019) 16:182–241. doi: 10.11909/j.issn.1671-5411.2019.03.014 PMC6500570 31080465

[18] Rantanen T Guralnik JM Foley D Masaki K Leveille S Curb JD. Midlife hand grip strength as a predictor of old age disability. JAMA. (1999) 281:558–60. doi: 10.1001/jama.281.6.558 10022113

[19] Storer TW Dolezal BA Berenc MN Timmins JE Cooper CB. Effect of supervised, Periodized exercise training vs. self-directed training on lean body mass and other fitness variables in health Club members. J Strength Cond Res. (2014) 28:1995–2006. doi: 10.1519/jsc.0000000000000331, PMID: 24276303

[20] Hansen D Abreu A Ambrosetti M Cornelissen V Gevaert A Kemps H. Exercise intensity assessment and prescription in cardiovascular rehabilitation and beyond: why and how: a position statement from the secondary prevention and rehabilitation section of the European Association of Preventive Cardiology. Eur. J Prev Cardiol. (2021) 29:230–45. doi: 10.1093/eurjpc/zwab007, PMID: 34077542

[21] Bull FC Al-Ansari SS Biddle S Borodulin K Buman MP Cardon G. World Health Organization 2020 guidelines on physical activity and sedentary behaviour. Br J Sports Med. (2020) 54:1451–62. doi: 10.1136/bjsports-2020-102955, PMID: 33239350 PMC7719906

[22] Sun X Su L Bu T Zhang Y. Exercise training upregulates intracellular nicotinamide phosphoribosyltransferase expression in humans: A systematic review with meta-analysis. Front Public Health. (2023) 11:11. doi: 10.3389/fpubh.2023.1287421, PMID: 37954044 PMC10639164

[23] Cheng P Wang L Ning P Peng Y Schwebel DC Liu J. Unintentional falls mortality in China, 2006-2016. J Glob Health. (2019) 9:010603. doi: 10.7189/jogh.09.010603, PMID: 30992985 PMC6445498

[24] Moreland B Kakara R Henry A. Trends in nonfatal falls and Fall-related injuries among Adults aged ≥65 years-United States, 2012-2018. MMWR Morb Mortal Wkly Rep. (2020) 69:875–81. doi: 10.15585/mmwr.mm6927a5, PMID: 32644982 PMC7732363

[25] Montero-Odasso M van der Velde N Martin FC Petrovic M Tan MP Ryg J. World guidelines for falls prevention and management for older adults: a global initiative. Age Ageing. (2022) 51:1–36. doi: 10.1093/ageing/afac205, PMID: 36178003 PMC9523684

[26] Shimbo D Bowling BC Levitan EB Deng L Sim JJ Huang L. Short-term risk of serious Fall injuries in older Adults initiating and intensifying treatment with antihypertensive medication. Circ Cardiovasc Qual Outcomes. (2016) 9:222–9. doi: 10.1161/CIRCOUTCOMES.115.002524, PMID: 27166208 PMC4871744

[27] Butt DA Mamdani M Austin PC Tu K Gomes T Glazier RH. The risk of falls on initiation of antihypertensive drugs in the elderly. Osteoporos Int. (2013) 24:2649–57. doi: 10.1007/s00198-013-2369-7, PMID: 23612794

[28] Tinetti ME Han L Lee DSH McAvay GJ Peduzzi P Gross CP. Antihypertensive medications and serious Fall injuries in a nationally representative sample of older Adults. JAMA Intern Med. (2014) 174:588–95. doi: 10.1001/jamainternmed.2013.14764, PMID: 24567036 PMC4136657

[29] Pescatello LS Franklin BA Fagard R Farquhar WB Kelley GA Ray CA. Exercise and hypertension. Med Sci Sports Exerc. (2004) 36:533–53. doi: 10.1249/01.Mss.0000115224.88514.3a 15076798

[30] Charchar FJ Prestes PR Mills C Ching SM Neupane D Marques FZ. Lifestyle management of hypertension: International Society of Hypertension position paper endorsed by the world hypertension league and European Society of Hypertension. J Hypertens. (2023) (In press). doi: 10.1097/hjh.0000000000003563 PMC10713007 37712135

[31] Saco-Ledo G Valenzuela PL Ruiz-Hurtado G Ruilope LM Lucia A. Exercise reduces ambulatory blood pressure in patients with hypertension: A systematic review and Meta-analysis of randomized controlled trials. J Am Heart Assoc. (2020) 9:e018487. doi: 10.1161/JAHA.120.018487, PMID: 33280503 PMC7955398

[32] Igarashi Y. Effects of differences in exercise programs with regular resistance training on resting blood pressure in hypertensive Adults: A systematic review and meta-analysis. J Strength Cond Res. (2023) 1:253–63. doi: 10.1519/jsc.0000000000004236, PMID: 35442242

[33] Baffour-Awuah B Pearson MJ Dieberg G Smart NA. Isometric resistance training to manage hypertension: systematic review and meta-analysis. Curr Hypertens Rep. (2023) 25:35–49. doi: 10.1007/s11906-023-01232-w, PMID: 36853479 PMC10014822

[34] Naci H Salcher-Konrad M Dias S Blum MR Sahoo SA Nunan D. How does exercise treatment compare with antihypertensive medications? A network meta-analysis of 391 randomised controlled trials assessing exercise and medication effects on systolic blood pressure. Br J Sports Med. (2019) 53:859–69. doi: 10.1136/bjsports-2018-099921, PMID: 30563873

[35] Baptista LC Amorim AP Valente-dos-Santos J Machado-Rodrigues AM Veríssimo MT Martins RA. Functional status improves in hypertensive older adults: the long-term effects of antihypertensive therapy combined with multicomponent exercise intervention. Aging Clin Exp Res. (2018) 30:1483–95. doi: 10.1007/s40520-018-0925-x, PMID: 29512042

[36] Ruangthai R Phoemsapthawee J. Combined exercise training improves blood pressure and antioxidant capacity in elderly individuals with hypertension. J Exerc Sci Fit. (2019) 17:67–76. doi: 10.1016/j.jesf.2019.03.001, PMID: 30949214 PMC6430041

[37] Lima LG Bonardi JTM Campos GO Bertani RF Scher LML Moriguti JC. Combined aerobic and resistance training: are there additional benefits for older hypertensive adults? Clinics. (2017) 72:363–9. doi: 10.6061/clinics/2017(06)06, PMID: 28658436 PMC5463253

[38] Coelho Júnior HJ Asano RY Gonçalvez IO Brietzke C Pires FO Aguiar SS. Multicomponent exercise decreases blood pressure, heart rate and double product in normotensive and hypertensive older patients with high blood pressure. Arch Cardiol Mex. (2018) 88:413–22. doi: 10.1016/j.acmx.2018.01.001, PMID: 29496407

[39] Leitão L Marocolo M de Souza HLR Arriel RA Campos Y Mazini M. One-year detraining effects after multicomponent exercise program in hypertensive older women. Int J Environ Res Public Health. (2022) 19:2871. doi: 10.3390/ijerph19052871, PMID: 35270564 PMC8910620

[40] Sardeli AV Gáspari AF dos Santos WM de Araujo AA de Angelis K Mariano LO. Comprehensive time-course effects of combined training on hypertensive older Adults: A randomized control trial. Int J Environ Res Public Health. (2022) 19:11042. doi: 10.3390/ijerph191711042, PMID: 36078774 PMC9518134

[41] dos Santos E Asano RY Filho IG Lopes NL Panelli P Nascimento Dda C. Acute and chronic cardiovascular response to 16 weeks of combined eccentric or traditional resistance and aerobic training in elderly hypertensive women: A randomized controlled trial. J Strength Cond Res. (2014) 28:3073–84. doi: 10.1519/jsc.0000000000000537, PMID: 24845208

[42] Vellas BJ Wayne SJ Romero L Baumgartner RN Rubenstein LZ Garry PJ. One-leg balance is an important predictor of injurious falls in older persons. J Am Geriatr Soc. (1997) 45:735–8. doi: 10.1111/j.1532-5415.1997.tb01479.x, PMID: 9180669

[43] Kojima G Masud T Kendrick D Morris R Gawler S Treml J. Does the timed up and go test predict future falls among British community-dwelling older people? Prospective cohort study nested within a randomised controlled trial. BMC Geriatr. (2015) 15:38. doi: 10.1186/s12877-015-0039-7, PMID: 25887660 PMC4403843

[44] Coelho Junior HJ Rodrigues B Feriani DJ Gonçalves IO Asano RY Aguiar SS. Effects of multicomponent exercise on functional and cognitive parameters of hypertensive patients: A quasi-experimental study. J Aging Res. (2017) 2017:1978670–10. doi: 10.1155/2017/1978670, PMID: 28409030 PMC5376403

[45] McGraw KO Wong SP. A common language effect size statistic. Psychol Bull. (1992) 111:361–5. doi: 10.1037/0033-2909.111.2.361

[46] Bullo V Gobbo S Vendramin B Duregon F Cugusi L Blasio AD. Nordic walking Can be incorporated in the exercise prescription to increase aerobic capacity, strength, and quality of life for elderly: A systematic review and Meta-analysis. Rejuvenation Res. (2018) 21:141–61. doi: 10.1089/rej.2017.1921, PMID: 28756746

[47] Wang J Xu J An R. Effectiveness of backward walking training on balance performance: A systematic review and meta-analysis. Gait Posture. (2019) 68:466–75. doi: 10.1016/j.gaitpost.2019.01.002, PMID: 30616175

[48] Lundin H Sääf M Strender LE Nyren S Johansson SE Salminen H. One-leg standing time and hip-fracture prediction. Osteoporos Int. (2014) 25:1305–11. doi: 10.1007/s00198-013-2593-1, PMID: 24562837

[49] Simek EM McPhate L Haines TP. Adherence to and efficacy of home exercise programs to prevent falls: A systematic review and meta-analysis of the impact of exercise program characteristics. Prev Med. (2012) 55:262–75. doi: 10.1016/j.ypmed.2012.07.007, PMID: 22813920

[50] Okubo Y Schoene D Lord SR. Step training improves reaction time, gait and balance and reduces falls in older people: a systematic review and meta-analysis. Br J Sports Med. (2017) 51:586–93. doi: 10.1136/bjsports-2015-095452, PMID: 26746905

[51] Moreno-Segura N Igual-Camacho C Ballester-Gil Y Blasco-Igual MC Blasco JM. The effects of the Pilates training method on balance and falls of older Adults: A systematic review and Meta-analysis of randomized controlled trials. J Aging Phys Act. (2018) 26:327–44. English. doi: 10.1123/japa.2017-0078, PMID: 28771109

[52] Shin S. Meta-analysis of the effect of yoga practice on physical fitness in the elderly. Int J Environ Res Public Health. (2021) 18:11663. doi: 10.3390/ijerph182111663, PMID: 34770176 PMC8583600

[53] Sherrington C Fairhall N Kwok W Wallbank G Tiedemann A Michaleff ZA. Evidence on physical activity and falls prevention for people aged 65+ years: systematic review to inform the WHO guidelines on physical activity and sedentary behaviour. Int J Behav Nutr Phys Act. (2020) 17:144. doi: 10.1186/s12966-020-01041-3, PMID: 33239019 PMC7689963

[54] Li F Harmer P Fitzgerald K Eckstrom E Stock R Galver J. Tai Chi and Postural stability in patients with Parkinson's disease. N Engl J Med. (2012) 366:511–9. doi: 10.1056/NEJMoa1107911, PMID: 22316445 PMC3285459

[55] Huang Y Liu X. Improvement of balance control ability and flexibility in the elderly Tai Chi Chuan (TCC) practitioners: A systematic review and meta-analysis. Arch Gerontol Geriatr. (2015) 60:233–8. doi: 10.1016/j.archger.2014.10.016, PMID: 25497683

[56] Huang Z-G Feng Y-H Li Y-H Lv C-S. Systematic review and meta-analysis: Tai Chi for preventing falls in older adults. BMJ Open. (2017) 7:e013661. doi: 10.1136/bmjopen-2016-013661, PMID: 28167744 PMC5293999

[57] Liang H Luo S Chen X Lu Y Liu Z Wei L. Effects of Tai Chi exercise on cardiovascular disease risk factors and quality of life in adults with essential hypertension: A meta-analysis. Heart Lung. (2020) 49:353–63. doi: 10.1016/j.hrtlng.2020.02.041, PMID: 32171586

[58] Ekkekakis P Parfitt G Petruzzello SJ. The pleasure and displeasure people feel when they exercise at different intensities. Sports Med. (2011) 41:641–71. doi: 10.2165/11590680-000000000-00000, PMID: 21780850

[59] Parfitt G Rose EA Burgess WM. The psychological and physiological responses of sedentary individuals to prescribed and preferred intensity exercise. Br J Health Psychol. (2006) 11:39–53. doi: 10.1348/135910705X43606 16480554

[60] Gearhardt AN Bueno NB DiFeliceantonio AG Roberto CA Jiménez-Murcia S Fernandez-Aranda F. Social, clinical, and policy implications of ultra-processed food addiction. BMJ. (2023) 383:e075354. doi: 10.1136/bmj-2023-075354, PMID: 37813420 PMC10561019

[61] Lee L-L Watson MC Mulvaney CA Tsai C-C Lo S-F. The effect of walking intervention on blood pressure control: A systematic review. Int J Nurs Stud. (2010) 47:1545–61. doi: 10.1016/j.ijnurstu.2010.08.008 20863494

[62] Lee L-L Arthur A Avis M. Evaluating a community-based walking intervention for hypertensive older people in Taiwan: A randomized controlled trial. Prev Med. (2007) 44:160–6. doi: 10.1016/j.ypmed.2006.09.001, PMID: 17055561

[63] Puthoff ML Janz KF Nielsen DH. The relationship between lower extremity strength and power to everyday walking behaviors in older Adults with functional limitations. J Geriatr Phys Ther. (2008) 31:24–31. doi: 10.1519/00139143-200831010-00005, PMID: 18489805

[64] Aranyavalai T Jalayondeja C Jalayondeja W Pichaiyongwongdee S Kaewkungwal J Laskin JJ. Association between walking 5000 step/day and fall incidence over six months in urban community-dwelling older people. BMC Geriatr. (2020) 20:194. doi: 10.1186/s12877-020-01582-z, PMID: 32503501 PMC7275504

[65] Paluch AE Bajpai S Bassett DR Carnethon MR Ekelund U Evenson KR. Daily steps and all-cause mortality: a meta-analysis of 15 international cohorts. Lancet Public Health. (2022) 7:e219–28. doi: 10.1016/S2468-2667(21)00302-9, PMID: 35247352 PMC9289978

[66] Kassaï B Boissel J-P Cucherat M Boutitie F Gueyffier F. Treatment of high blood pressure and gain in event-free life expectancy. Vasc Health Risk Manag. (2005) 1:163–9. doi: 10.2147/vhrm.s1216364086, PMID: 17315403 PMC1993937

[67] Bai X Xiao W Soh KG Agudamu ZY. 12-week concurrent brisk walking and Taijiquan (Tai Chi) improve balance, flexibility, and muscular strength of Chinese older women. PLoS One. (2023) 18:e0293483. doi: 10.1371/journal.pone.0293483, PMID: 37883372 PMC10602331

[68] Oja P Kelly P Pedisic Z Titze S Bauman A Foster C. Associations of specific types of sports and exercise with all-cause and cardiovascular-disease mortality: a cohort study of 80 306 British adults. Br J Sports Med. (2017) 51:812–7. doi: 10.1136/bjsports-2016-096822, PMID: 27895075

[69] Martens CR Denman BA Mazzo MR Armstrong ML Reisdorph N McQueen MB. Chronic nicotinamide riboside supplementation is well-tolerated and elevates NAD+ in healthy middle-aged and older adults. Nat Commun. (2018) 9:1286. doi: 10.1038/s41467-018-03421-7, PMID: 29599478 PMC5876407

[70] Coventry PA Brown JE Pervin J Brabyn S Pateman R Breedvelt J. Nature-based outdoor activities for mental and physical health: systematic review and meta-analysis. SSM Popul Health. (2021) 16:100934. doi: 10.1016/j.ssmph.2021.100934, PMID: 34646931 PMC8498096

[71] Li J Wan B Yao Y Bu T Li P Zhang Y. Chinese path to sports modernization: fitness-for-all (Chinese) and a development model for developing countries. Sustainability. (2023) 15:4203. doi: 10.3390/su15054203

[72] Williams DM. Exercise, affect, and adherence: An integrated model and a case for self-paced exercise. J Sport Exerc Psychol. (2008) 30:471–96. English. doi: 10.1123/jsep.30.5.471, PMID: 18971508 PMC4222174

